# Using theories of behaviour change to transition multidisciplinary trauma team training from the training environment to clinical practice

**DOI:** 10.1186/s13012-019-0890-6

**Published:** 2019-04-29

**Authors:** Margaret Murphy, Andrea McCloughen, Kate Curtis

**Affiliations:** 1 Sydney Nursing School, University of Sydney, Sydney, Australia; 2 Emergency Department, Westmead Hospital, Hawkesbury Road, Westmead, NSW 2145 Australia; 3 Emergency Department, Illawarra Shoalhaven Local Health District, Nowra, Australia

**Keywords:** Trauma resuscitation team, Simulation, Multidisciplinary team training, Implementation science, Theoretical domains framework

## Abstract

**Background:**

Major trauma patients—such as patients who have experienced road injury, high-impact falls or violence—require complex, intense and rapid resuscitation from a multidisciplinary team of clinicians. These ‘flash teams’ must form quickly and function effectively, often having never met before. There is evidence that multidisciplinary teamwork training improves the performance of the trauma team in simulation. However, the translation of learnt resuscitation teamwork skills from simulation into clinical practice has had modest and variable effects. This paper outlines a method for developing an intervention designed to translate the teaching from a simulated training environment into clinical practice using the theoretical domains framework, behaviour change wheel and behaviour change techniques as the theoretical and empirical basis for the process.

**Methods:**

The data used to inform the intervention development process were collected during an implementation evaluation study of the trauma team training programme at the busiest level 1 trauma centre in Sydney, Australia. A detailed barrier and enabler assessment were conducted using qualitative and quantitative data. The theoretical domains framework was used to integrate the results. Implementation interventions were selected using the behaviour change wheel.

**Results:**

Twenty-three facilitators and 19 barriers were identified to influence the implementation of trauma team training in the clinical setting. The facilitators and barriers corresponded to all 14 domains of the theoretical domains framework. Seven intervention functions and four policy categories of the behavioural change wheel were selected to address the target behaviours, and a multimodal relaunch of the revised trauma team training programme was developed.

**Conclusions:**

This study offers a framework for deductively employing the theoretical domains framework, behaviour change wheel and behaviour change techniques to assess and develop intervention strategies to improve the functioning of trauma resuscitation teams.

## Background

Major trauma patients, such as road injury, high impact falls and violence require complex, intense and rapid management in specialised trauma centres. These centres have the resources and expertise to provide care for critically injured patients. Within the trauma centre is the trauma team [1]. Such teams usually comprise a multidisciplinary group of individuals from the specialties of emergency medicine, trauma, intensive care, surgery, nursing, radiology, allied health and support staff. The team is activated when a patient meets the trauma call criteria, which includes a mechanism of injury such as fall, assault or motor vehicle collision greater than 60kph with associated clinical or injury criteria. When the trauma team is activated, the individual speciality members assigned to the trauma team are required to attend the trauma patient in the Emergency Department (ED) as soon as possible. Therefore, the trauma team is often being formed as the clinical situation develops. Team members may not have met each other or worked together previously. Trauma teams are consequently ‘flash’ teams with predetermined team roles but constantly changing membership.

Trauma teams’ function in complex environments that involve complicated decision-making and time pressures with serious implications if errors are made. In this environment, the trauma team is required to resuscitate, diagnose and treat critically injured patients [2]. For trauma teams to be effective, multidisciplinary team skills are required [3–5]. However, teamwork does not occur spontaneously; like all other skills, it must be learned. Efforts to support teamwork in trauma resuscitation teams include simulation-based team training with a focus on teaching non-technical skills [6]. This training improves the performance of the team in simulation, but there are challenges translating the knowledge into practice [7, 8]. The literature describing the knowledge translation from the simulation training environment into actual clinical practice is scarce [6, 9]. There is little known about how this training is used in resuscitation practice, in particular team members’ opinions on ease of use, behaviours driving the team’s success and applicability to the fast-moving work environment of a resuscitation bay. Further, the challenges encountered by the trauma team in an actual trauma resuscitation differ significantly from the challenges experienced in simulated training.

The successful implementation of any new training into the health care system require clinicians to change their behaviour [10]. This is central to the successful translation of new knowledge to clinical practice [11]. But changing behaviour is difficult and multifaceted. Implementing practice change in trauma resuscitation teams is particularly complex as it requires changes in individual and collective behaviour due to the diversity of disciplines and specialties involved. Consideration of the influences on behaviour in the setting in which they occur is required [12]. It is necessary to understand the facilitators and barriers to behaviour change in order to select the appropriate intervention required to promote the uptake and sustained use of the new ways of working [13].

The transition of educational initiatives and innovation into clinical practice is a slow and difficult process to navigate and can take years [14, 15]. Theoretical methods have had more success in achieving change than non-theory-driven models [13, 16, 17]. Theory can be used to identify the clinical behaviour being targeted for change, to find techniques to modify it and to describe how change interventions might work. To date, we have not identified any theory-based implementation strategy that has tackled promoting uptake and application of teamwork training to real life trauma resuscitation practice. This paper outlines a theory-informed strategy to implement a revised multidisciplinary trauma team training (TTT) programme into clinical practice. It uses the theoretical domains framework (TDF), the behaviour change wheel and behaviour change techniques [18] as the theoretical and empirical basis to transition from training to clinical practice.

## Aims

The primary aim of this paper is to identify facilitators and barriers to the implementation of a revised trauma team training programme into clinical practice. The secondary aim is to design strategies to translate the educational outcomes into clinical practice.

## Method

The study to be addressed in this paper is the development of an implementation strategy for a revised multidisciplinary TTT programme. The original TTT was developed at an adult trauma hospital in Sydney, Australia, where over 75,000 emergency and 4250 trauma patients present annually. Its aim was to train the multidisciplinary trauma team in teamwork. That training programme commenced in 2009.

**Fig. 1:**
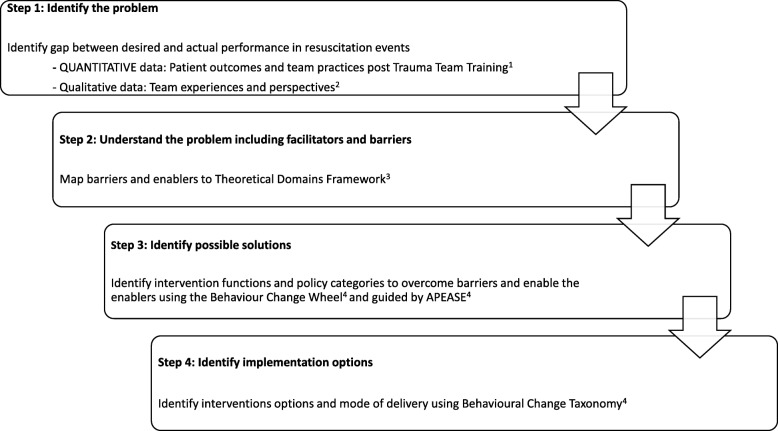
Overview of study design to develop a theory-informed implementation intervention for a multidisciplinary trauma team training programme. 1. Murphy M, Curtis K, Lam MK, Palmer CS, Hsu J, McCloughen A. Simulation-based multidisciplinary team training decreases time to critical operations for trauma patients. Injury. 2018;49 (5):953–958. 2. Murphy M, McCloughen A and Curtis K. The impact of simulated multidisciplinary Trauma Team Training on team performance: A qualitative study. Australasian Emergency Care. 10.1016/j.auec.2018.11.003. 3. French SD, Green SE, O’Connor DA, et al. Developing theory-informed behaviour change interventions to implement evidence into practice: a systematic approach using the Theoretical Domains Framework. Implementation Science. 2012;7 (1):38. 4. Michie S, van Stralen M, West R. The Behaviour Change Wheel: a new method for characterising and designing behaviour change interventions. Vol 6; 2011

A four-step approach to designing an implementation intervention was used (Fig. 1): (1) identify the problem, (2) evaluate the problem and identify which barriers and enablers need to be changed, (3) design possible solutions to modify barriers and enhance enablers and (4) operationalise the behaviour change solutions.

### Step 1: Identify the problem

A mixed methods implementation evaluation study was used to obtain a clear and accurate understanding of current trauma practices, processes and outcomes following the original TTT programme. Three methods were used: the influence of TTT on patient outcomes and health service delivery [8], facilitators and enablers to the clinical application of teamwork skills taught in TTT [19] and trauma team members’ experiences and perspectives of teamwork after participating in TTT [20].

### Data collection

#### Impact of TTT on patient outcomes and health service delivery

A retrospective review of trauma registry data was conducted. Inclusion criteria were adult patients admitted to the hospital (aged > 18 years old) with major traumatic injury (Injury Severity Score > 12 [21]. Demographic information including age and gender as well as clinical information including Injury Severity Score (ISS), time to critical interventions, ED length of stay (LOS) and mortality was collected. Two concurrent 4-year periods, before and after implementation of the original TTT programme, were compared for differences in time to critical operations, ED LOS and patient mortality. There were 2389 major trauma patients admitted to the study, 1116 in the 4 years preceding trauma team training (the PRE-group) and 1273 in the subsequent 4 years (the POST group).

#### Facilitators and barriers to use of teamwork skills

A survey was conducted in October and November 2016, using a 38-item survey. Eligibility to complete the survey included participants having completed TTT and being currently employed at the study site as members of the trauma team. Three hundred forty-five clinicians had completed TTT but 110 no longer worked at the study site. Two hundred thirty-five clinicians were emailed an invitation to participate with a link to the online survey. To identify implementation problems, the survey was designed and mapped to the TDF [22]. It included questions on the impact of training on the practice of teamwork by the trauma team when resuscitating critically injured patients in clinical practice.

#### Trauma team members’ experiences and perspectives on teamwork following TTT

Interviews were conducted in December 2016. Trauma team members were identified by indicating their willingness to participate in a follow-up interview when completing the survey during the quantitative phase of the study. Participants were encouraged to share their perspectives on current team performance and to reflect on their practice as a trauma team member since completing the training. Fifteen participants were interviewed. The interviews took on average 42 min to complete. They were audio recorded and later transcribed verbatim.

### Ethics

Approval to conduct the study was granted from the Local Health District Human Research Ethics Committee Reference (4199) LNR/15/WMEAD/18. Consent from staff was implied by survey completion, and written consent to interview was obtained.

### Data analysis

#### Impact of TTT on patient outcomes and health service delivery

Comparisons of patient outcomes, before and after implementation of TTT, were reviewed for differences in time to critical operations and patient mortality using the chi-square test and the Mann–Whitney *U* test.

#### Facilitators and barriers to use of teamwork skills

Quantitative data obtained from study surveys were imported to SPSS (IBM V 23) [23] and analysed using descriptive statistics.

#### Trauma team members’ experiences and perspectives on teamwork following TTT

Qualitative data obtained from interviews were imported into excel. Descriptive codes were examined for patterns, collated and summarised. Thirty-four initial codes were developed. Coded data were then interrogated more deeply, and six candidate themes were developed. These themes were further refined to four core themes.

### Results

#### Impact of TTT on patient outcomes and health service delivery

There were no differences between the groups with respect to gender, body region injured, incidence of polytrauma and pattern of arrival to ED. The POST group was older (median age 54 versus 43 years, *p* < 0.001) and had a higher incidence of falls and assaults (*p* < 0.001). There was a reduction in time to critical operation, from 2.63 h (IQR 1.23–5.12) in the PRE-group to 0.55 h (IQR 0.22–1.27) in the POST-group, *p* < 0.001. The overall ED LOS increased and there was no reduction in mortality. This prolonged length of stay is likely attributed to an increase in ED presentations without a comparative growth in the hospital’s bed capacity, resulting in ED overcrowding and congestion [8]. Post hoc analysis found LOS in ED was reduced in the cohort requiring critical operations, *p* < 0.001. Other than an increase in the number of Emergency Consultants employed to provide weekend cover for all emergency patients, there were no other significant changes identified during the study period.

#### Facilitators and barriers to use of teamwork skills

Of 235 eligible participants, 86 responded (rate 37%). All professional groups and clinical services were represented; about half of respondents were nurses (53%, *n* = 44) followed by doctors (43%, *n* = 37). There were 16 facilitators and 12 barriers to the use of teamwork skills in trauma resuscitation identified. Barriers and facilitators were allocated to categories of factors known to influence trauma team practices. These categories were (1) organisational factors that influence the trauma team, (2) team factors that influence teamwork and (3) cognitive factors that influence team decision-making.

#### Trauma team members’ experiences and perspectives on teamwork following TTT

Twelve females (*n* = 12) and three males (*n* = 3) participated in the interviews. All professional groups were represented: nurses (40.0%, *n* = 6), doctors (40.0%, *n* = 6) and allied health staff (20.0%, *n* = 3). All disciplines were represented with the majority represented by ED (46.6%, *n* = 7), followed by Trauma Service (20.0%, *n* = 3), Anaesthetic Service (13.3%, *n* = 2), Radiology (13.3%, *n* = 2) and Social Work (6.6.3%, *n* = 1). Four themes were developed: Leader-follower synergy promotes trauma teamwork, instability and inconsistency threaten trauma teamwork, clear communication enhances trauma team decision-making and team training improves trauma team performance.

**Fig. 2:**
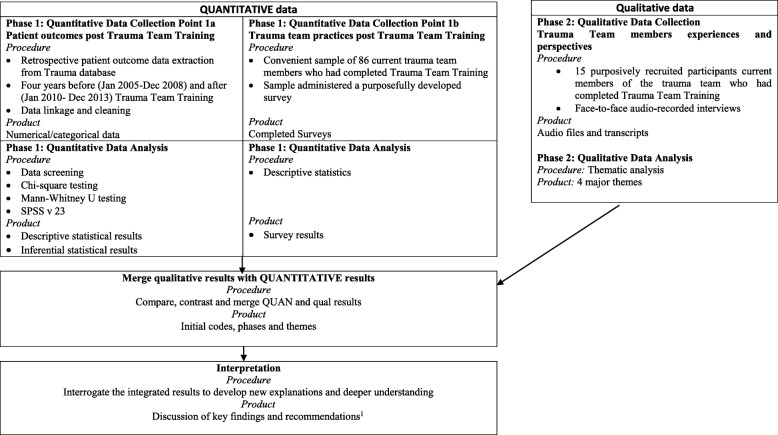
Visual model of a mixed methods study to evaluate the training of trauma resuscitation flash teams. 1. Murphy M, McCloughen A, Curtis K. Enhancing the training of trauma resuscitation flash teams: a mixed methods study. *Australasian Emergency Care.* 2018;21 (4):143–149

A visual model of how the quantitative and qualitative data were collected, analysed separately and then merged is displayed in Fig. 2 and has been reported previously [24]. This data were used to identify the barriers and facilitators to TTT implementation into clinical practice. These themes were mapped against 14 of the TDF domains (Table 1).

**Table 1:** Facilitators and barriers, linked to the theoretical domains framework, illustrated by qualitative and quantitative results

TDF domain	Factors affecting implementation	Facilitator (F) Barrier (B)	Sources *Team Survey data n = 86* *Team interview data n = 15* *Patient outcome data n = 2389*	Questions/sample quote/patient outcome data					Result
Knowledge (an awareness of the existence of something)	Not knowing what to do to activate a major trauma call	B	Team survey (Descriptive statistics)	Do you know how to activate a major trauma call?					
				a. All the time					66.3% (*n* = 53)
				b. Sometimes					3.5% (*n* = 4)
				c. Never					30.2% (*n* = 26)
	Unaware of team members roles	B	Team survey (Descriptive statistics)	Are you aware of other team member’s activities during a major trauma?					
				a. All the time					72.1% (*n* = 62)
				b. Sometimes					25.6% (*n* = 22)
				c. Never					2.3% (*n* = 2.3)
	Knowledge of trauma system	F	Team interview (Thematic analysis)	Everyone now knows how the system works and where they fit into it					
Skills (an ability or proficiency required through practice)	Non-technical skill of closed loop communication not used	B	Team survey (Descriptive statistics)	Team members practise closed loop communication					
				a. All the time					59.3% (*n* = 51)
				b. Sometimes					37.2% (*n* = 32)
				c. Never					3.5% (*n* = 3)
	Multidisciplinary training is needed if you work clinically in a multidisciplinary team	F	Team interview (Thematic analysis)	*There is no point in doctors and nurses’ training separately as we are treating together*					
	Trauma Team Training teaches non-technical skills	F	Team interview (Thematic analysis)	*I found the teaching of the non-technical skills most beneficial*					
	The skills learnt in simulation trauma team training are not used in real life resuscitation	B	Team interview (Thematic analysis)	*The simulation environment is not realistic as it does not deal with the reality of multiple traumas and the impact this has on teamwork*					
Social/professional role and identity (a coherent set of behaviours and displayed social qualities of an individual in a social or work setting)	Team hierarchy	B	Team interview (Thematic analysis)	*I think we have a power gradient and if we could break this down I think our team would function better*					
	Professional disharmony	B	Team interview (Thematic analysis)	*Some doctors rather their opinion over a nurse no matter how much you train and work with them*					
	Professional role unclear	B		Trauma team members know each other’s roles and responsibilities					
				a. All the time					69.6% (*n* = 60)
				b. Sometimes					26.7% (*n* = 23)
				c. Never					3.5% (*n* = 3)
Beliefs about capabilities (professional confidence, beliefs, self-confidence, self-esteem, empowerment)	Perception that clinical judgement is better than cognitive aids in resuscitation practice	B	Team survey (Descriptive statistics)	Team members use cognitive aids to assist trauma management					
				a. All the time					53.5% (*n* = 46)
				b. Sometimes					38.4% (*n* = 33)
				c. Never					8.1% (*n* = 7)
	Team members are empowered to speak up	F	Team interview (Thematic analysis)	E*veryone seems comfortable to ask for clarification or question what is going o*n					
Belief about consequences (belief, outcome expectancies, consequences)	Standardised operating procedures assist trauma service delivery	F	Team survey (Descriptive statistics)	Prior planning and preparation assist the team to manage a major trauma.					
				a. All the time					91.9% (*n* = 77)
				b. Sometimes					5.8% (*n* = 5)
				c. Never					2.3% (*n* = 2)
	Standardising trauma care optimises team performance.	F	Team interview (Thematic analysis)	*Everyone knowing their roles made the resus run smoothly and the team were faster to assess and treat the patient.*					
	Leader-follower synergy promotes team work	F	Team interview (Thematic analysis)	*You need to include all parties as a team leader by themselves means nothing*					
Motivation and goals (mental representations of outcomes or end states that an individual wants to achieve, e.g. intention, goals, target setting, action planning, goal priority)	The team does not know the management plan as it evolves.	B	Team survey (Descriptive statistics)	The team leader updates the team by recapping the treatment plan					
				a. All the time					60.5% (*n* = 52)
				b. Sometimes					37.2% (*n* = 32)
				c. Never					2.3% (*n* = 2
	Shared mental models are used by the trauma team.	F	Team interview (Thematic analysis)	*The treatment goal is established and we all work to achieve that goal*					
	Time to critical operation and mortality was reduced	F	Patient outcome data (Pre-post study)	ED to Critical Operation (hrs)	n	Pre Median (IQR)	n	Post Median (IQR)	
					141	2.63 (1.23–5.12)	149	0.55 (0.22–1.27)	
				Mortality:		%		%	
				Died	35	24.80%	25	16.80%	
				Survived	106	75.20%	124	83.20%	
Memory, attention and decision processes (decision-making, cognitive overload, attention control, memory)	Shared decision-making	F	Team survey (Descriptive statistics)	Decisions are made with input and shared knowledge from team member					
				a. All the time					72.1% (*n* = 62)
				b. Sometimes					23.3% (*n* = 20)
				c. Never					4.7% (*n* = 4)
	Collaboration is needed to enhanced team decision-making	B	Team interview (Thematic analysis)	*It was one of the most poorly managed traumas and I was so upset because they would not listen to us and made the wrong decision*					
Environmental context and resources (environmental stressor, resources, salient events, organisational culture)	Environment is prepared	F	Team survey (Descriptive statistics)	Is the resuscitation equipment checked and assembled as needed?					
				a. All the time					90.7% (*n* = 78)
				b. Sometimes					7.0% (*n* = 6)
				c. Never					2.3% (*n* = 2
	Relevant resources are notified	F	Team survey (Descriptive statistics)	Are relevant support staff and services notified (radiology, blood bank) notified?					
				a. All the time					80.2% (*n* = 69)
				b. Sometimes					17.5% (*n* = 15)
				*c.* Never					2.3% (*n* = 2)
	Noise and chaos in a resuscitation impacts patient safety	B	Team interview (Thematic analysis)	*When it’s a big mess with too many people and too much noise, we are too slow and you end up missing the boat with this patient*					
	Trauma team has constantly changing membership	B	Team interview (Thematic analysis)	*One of the challenges is the highly mobile workforce that keep changing so the skills of the team keep changing*					
Optimism (the confidence that things will happen for the best or the desired goals will be attained)	There is a prompt response when the trauma team is activated	F	Team survey (Descriptive statistics)	On activation of a major trauma call do team members attend promptly?					
				a. All the time					80.2% (*n* = 69)
				b. Sometimes					16.3% (*n* = 14)
				c. Never					3.5% (*n* = 3)
				The size and composition of the trauma team is appropriate for managing major trauma.					
	The trauma team was the right size to assist service delivery	F	Team survey (Descriptive statistics)	a. All the time					82.5% (*n* = 71)
				b. Sometimes					14.0% (*n* = 12)
				c. Never					3.5% (*n* = 3
	Better care is now provided as the team is co-ordinated.	F	Team interview (Thematic analysis)	*A co-ordinated team response has got to be better for the patient as they get immediate assessment, then everything is reviewed and titrated to their clinical needs*					
Social influences (those interpersonal processes that can cause individuals to change their thoughts, feelings and behaviours)	Team is confident to question decisions made by team leader	F	Team survey (Descriptive statistics	Is there is a reluctance to question decisions or actions of a senior doctor/team leader during a trauma?					
				a. All the time					15.1% (*n* = 13)
				b. Sometimes					80.2% (*n* = 69)
				c. Never					4.7% (*n* = 4)
	Poor conflict resolution	B	Team survey (Descriptive statistics)	The team leader discusses areas of concern/conflict with the team and explains rationale for decisions made					
				a. All the time					52.3% (*n* = 45)
				b. Sometimes					33.7% (*n*-29)
				c. Never					13.9% (*n* = 12)
	Team members do not know each other	B	Team survey (Descriptive statistics	Do trauma team members know each other’s roles and responsibilities?					
				a. All the time					69.6% (*n* = 60)
				b. Sometimes					26.7% (*n* = 23)
				c. Never					3.5% (*n* = 3)
	Not confidence to speak up	B	Team survey (Descriptive statistics	I am confident to ‘speak up’ to communicate a problem to other members of the team					
				a. All the time					51.2% (*n* = 44)
				b. Sometimes					47.6% (*n* = 41)
				c. Never					1.2% (*n* = 1)
	Psychological safety enhanced communication	F	Team interview (Thematic analysis)	*found it helpful as it gave ou permission to talk in a way that previously would have been viewed as touchy feely*					
	Escalation ignored by medical staff	B	Team interview (Thematic analysis	*I remember thinking you know us, we do not make a fuss unless we are concerned so why are you not listening to us and they end up making poor decisions*					
	Team conflict impacts patient care	B	Team interview (Thematic analysis)	*Everyone managing this resusitation trauma was involved in a previous one where there was conflict in the team and I feel this conflict continued in this resusitation.*					
Emotion (fear, anxiety, affect, stress, dealing with a personally significant matter/event)	Lack of trust and respect	B	Team survey (Descriptive statistics)	Is there mutual respect and trust between trauma team members?					
				a. All the time					69.8% (*n* = 60)
				b. Sometimes					24.4% (*n* = 21)
				c. Never					5.8% (*n* = 5)
		B	Team interview (Thematic analysis)	*I observe that when people are not familiar with each other there is tension and conflict and this affects the team*					
Behavioural regulation (anything aimed at managing or changing objectively observed or measured actions, i.e. self-monitoring, action planning)	There is compliance with nonverbal communication techniques	F	Team survey (Descriptive statistics	Are coloured role tags worn by each team member?					
				a. All the time					72.1% (*n* = 62)
				b. Sometimes					26.7% (*n* = 23)
				c. Never					1.2% (*n* = 1)
	Procedural compliance	F	Team survey (Descriptive statistics)	Is the pre-notification handover displayed on board in resus?					
				a. All the time					90.7% (*n* = 78)
				b. Sometimes					7.0% (*n* = 6)
				c. Never					2.0% (*n* = 2)
	Teamwork is practised in real life resuscitations.	F	Team interview (Thematic analysis)	*Yes, I do see teamwork being practised by the doctors and nurses*					
Reinforcement (increasing the probability of a response by arranging a dependant relationship between the response and a given stimulus)	Standardisation of operating procedures helps to reinforce team members’ roles and responsibilities.	F	Team survey (Descriptive statistics)	The Team Leader identifies him/herself to the treating paramedic					
				a. All the time					75.6% (*n* = 65)
				b. Sometimes					22.1% (*n* = 19)
				c. Never					2.3% (*n* = 2)
	Reminders	F	Team interview (Thematic analysis)	*The nursing team leader is very good at prompting me when I forget things*					
Intentions (a conscious decision to perform a behaviour or a resolve to act in a certain way)	The team leader leads	F	F Team survey (Descriptive statistic	The team leader maintains a ‘hands free’ approach to leading the trauma.					
				a. All the time					86.0% (*n* = 74)
				b. Sometimes					10.5% (*n* = 9)
				c. Never					3.5% (*n* = 3)

## Solution design

Having established what needed to change, the behaviour change wheel (BCW) [18] was used to design interventions to improve implementation of TTT in clinical practice. TDF domains recognised to be targets of change were plotted against the BCW. This process is outlined in Table 2. Intervention functions of the BCW were linked to the behaviour change techniques taxonomy [18]. Prior to selecting interventions, it was necessary to determine which intervention functions, policy categories, behaviour change techniques and modes of delivery would be most appropriate for the trauma context and therefore most likely to be implemented and have an impact. To do this, the experience of the research and education team, together with feedback from managers and clinical colleagues, was used. A series of focus groups were held with the research team, simulation educators and frontline staff to consider the modes of delivering interventions before deciding the most appropriate for the trauma team in the resuscitation setting. The APEASE criteria were used [18]. This criterion outlined the dimensions to consider in selecting interventions and included affordability, practicality, effectiveness and cost-effectiveness, acceptability, side-effects/safety and equity. In selecting the preferred modes of delivery, the cost was considered including the cost of releasing staff to attend training. Practical considerations were addressed such as can the revised TTT be delivered as designed to the all multidisciplinary team members. Data highlighted the positive outcomes from TTT. The acceptability of TTT as a workforce training strategy was discussed with managers and educators. The proposed intervention functions were reviewed for unwanted side-effects or safety consequences. Equity for team members and patients was also considered.

**Table 2:** The 14 TDF domains identified to contain facilitators and barriers (vertical) mapped to intervention functions (horizontal)

	Education	Training	Restriction	Environmental restructuring	Modelling	Enablement	Persuasion	Coercion	Incentivisation
Knowledge	✓								
Skills	✓	✓							
Social/professional role and identity	✓				✓		✓		
Beliefs about capabilities	✓				✓	✓	✓		
Belief about consequences	✓				✓	✓	✓		
Motivation and goals	✓				✓	✓	✓	✓	✓
Memory, attention and decision processes		✓		✓		✓			
Environmental context and resources		✓	✓	✓		✓			
Optimism	✓				✓	✓	✓		
Social influences			✓	✓	✓	✓			
Emotion					✓	✓	✓	✓	✓
Behavioural regulation	✓				✓	✓			
Reinforcement		✓			✓	✓		✓	✓
Intentions	✓	✓							

## Operationalising the behaviour change solutions

The behaviour change methodology described above was used to inform the development and design of interventions to improve the implementation of the revised TTT. These proposed implementation strategies are outlined in Table 3 and discussed in the text below.

**Table 3:** Behaviour change techniques (BCTs), modes and content of delivery to implement the seven prioritised interventions for multidisciplinary trauma team training

Interventions functions	Which BCTs could overcome the modifiable barriers and enhance the enablers.	Proposed intervention components (how the techniques will be delivered and what content will be delivered)
Facilitators and barriers relating to educational strategies for training a ‘flash’ trauma resuscitation team		
Education Environmental restructuring Persuasion Modelling Enablement	Information about consequences Instructions on performing the behaviour Feedback on behaviour Problem-solving Action planning Social support Demonstration of the behaviour Social influences	Trauma Team Training programme will be modified to include: 1. Train ‘flash teams’ in ‘teaming’ which is teamwork on the fly^a^ 2. Contextualised simulated team training^b^ 3. Spaced learning^b^ 4. Rapid cycle deliberate practice of non-technical skills^c^
Facilitators and barriers relating to moving from the concept of a ‘Team’ to ‘Teaming’		
Education Training Persuasion Enablement	Policy category Regulation Guidelines Service Provision Communication/marketing	1. Regulations: Trauma governance committee 2. Guidelines: Trauma algorithms (treatment protocols) 3. Communication plan: TTT multi-media campaign 4. Service Provision: All trauma team members complete TTT
Facilitators and barriers relating to team culture in a ‘flash’ trauma resuscitation team		
Modelling Enablement Incentivisation Environmental restructuring	Credible source Information about others approval Demonstration of the behaviour Salience of consequences Commitment Verbal persuasion about capacity Social support Social reward Goal setting Review behaviour goals	1. Sponsorship from senior leaders and managers 2. Team leader buddy system
Facilitators and barriers to standardising operational procedures to enable co-ordination in ‘flash’ trauma resuscitation teams		
Environmental restructuring Modelling Enablement Persuasion Incentivisation	Adding objects to the environment Prompts/cues Restructuring the physical environment Demonstration of the behaviour Salience of consequences Information about consequences Commitment Feedback on outcomes of the behaviour Goal setting Social rewards Social support	1. Checklist and cognitive aids 2. Revision of equipment organisation and ergonomics 3. A stop clock to time-critical events/processes 4. Structure teamwork prompts, e.g. Zero-point survey^d^ 5. Structured debriefing tool^e^

^a^Edmondson AC. Teamwork on the fly. *Harvard Business Review.* 2012;90 (4):72–80

^b^Cheng A, Nadkarni VM, Mancini MB, et al. Resuscitation Education Science: Educational Strategies to Improve Outcomes From Cardiac Arrest: A Scientific Statement From the American Heart Association. *Circulation.* 2018:CIR. 0000000000000583

^c^Burden AR, Pukenas EW, Deal ER, et al. Using Simulation Education With Deliberate Practice to Teach Leadership and Resource Management Skills to Senior Resident Code Leaders. *Journal of Graduate Medical Education.* 2014;6 (3):463–469

^d^Reid C, Brindley P, Hicks C, et al. Zero point survey: a multidisciplinary idea to STEP UP resuscitation effectiveness. *Clin Exp Emerg Med.* 9 2018;5 (3):139–143

^e^Rose S, Cheng A. Charge nurse facilitated clinical debriefing in the emergency department. *Canadian Journal of Emergency Medicine.* 2018:1–5

### Step 1: What needs to change?

The target behaviour for the implementation of the revised TTT were as follows: (1) design education strategies specific to training a ‘flash’ trauma resuscitation team, (2) train the ‘flash’ team in ‘teaming’ which is teamwork specific to a spontaneously created team with constantly changing membership, (3) build a culture of psychological safety within the ‘flash’ team and (4) standardise resuscitation procedures and optimise environmental readiness. These target behaviours were chosen because they had supporting evidence from our study, were potentially modifiable and were behaviours to be performed by the trauma team during resuscitation.

### Step 2: Using the TDF, identify which barriers and enablers need to be addressed

To guide the development of interventions, we used the TDF to identify the barriers and enablers to the target behaviours and to choose the interventions. Twenty-three (23) facilitators and nineteen (19) barriers were identified. The facilitators and barriers corresponded to all 14 domains of the TDF. These facilitators and barriers were linked to the target behaviours; some are described further here, and all are outlined in Table 1 where data sources of the identified factors are presented and illustrative quotes are provided.

#### Skills

Lack of a common language across the trauma team was a barrier to teamwork. Survey respondents supported the concept of team training as a way to improve multidisciplinary teamwork and found the teaching of teamwork in simulation most beneficial. However, even though these skills were appreciated, they were not explicitly practised in the setting of a resuscitation. The fidelity of the simulation environment was identified as a reason for this as the qualitative data indicated that challenges encountered by the team in the emergency resuscitations differed from challenges experienced during simulated training.

#### Social/professional role and identity

Conflicting social/professional role influences were evident. These included not knowing the team, (identity and professional role) illustrated by almost 70% of survey respondents stating that they did not know team members’ roles and responsibilities. Participants also struggled to share the same mental models. During resuscitation, it was not clear how the team would work together. Interview participants provided insight into another potential social/professional barrier to teamwork in practice—the professional team culture where members tended to focus on their individual performance instead of adopting a team mindset. This affected teamwork as team members were reluctant to speak up, ask for help or admit error. Power gradients were perceived which limited sharing of information between team members and this hampered teamwork. Engagement between different specialities within the team was varied. It was challenging for some professionals to respond to the expectations of them outside their speciality environment. Functioning as a member of a larger trauma team, comprising of various disciplines and specialities, was different from working in their speciality team where they had long-standing working relationships. Participants perceived this lack of familiarity between team members as a catalyst for poor teamwork in real-world resuscitations.

#### Beliefs about capabilities

Only fifty-three (53%) percent of respondents used cognitive aids (such as trauma algorithms or clinical pathways) to assist trauma management. Survey respondents perceived clinical judgement to be better than cognitive aids as a decision-making strategy in resuscitation practice. This reliance on cognitive appraisal to resuscitate a critically injured patient is not supported by the literature. Optimal trauma care supports the use of mnemonic, clinical pathways and safety checklists in critical resuscitations to reduce the cognitive load associated with deliberation and problem solving [25, 26]. Despite the TTT programme teaching that trauma algorithms were safer to use, the extra steps to do something different in an emergency may imply it is easier to revert to previous familiar practice. Interview participants indicated they were positive about their ability to speak up to ask for clarification during a resuscitation. This is important in a ‘flash’ resuscitation team as it allows team leaders to incorporate multiple perspectives and tap into the knowledge of individual members.

#### Motivation and goals

When the treatment goal was established in a resuscitation by the trauma medical team leader and shared with the entire team, the participants stated that it helped them to achieve time-critical interventions. It optimised team performance and promoted the team’s ability to adapt and respond to dynamic or unpredictable events. This was reflected in patient outcomes with a reduction in time to critical operations following the training. However, participants stated that only 60% of team leaders summarised the treatment plan which illustrates that team leaders also struggle to use non-technical skills when leading a resuscitation.

#### Environmental context and resources

Two factors that negatively influence implementation were highlighted. Firstly, the constantly changing membership of the team was perceived as a threat to the retention of teamwork skills. Participants were unsure of who was in their team due to the changing composition of the team from shift to shift and sometimes from patient to patient. This instability complicated team processes and threatened teamwork because it was difficult for members to anticipate each other’s skill, knowledge and experience. Secondly, exposure to excessive noise and chaos caused by overcrowding in the resuscitation bay hampered their ability to provide safe patient care. However, survey respondents reported that equipment was prepared and relevant support services were notified which optimised resuscitation efficiency.

#### Social influences

Several conflicting social influences were evident. These centred on team dynamics and team culture. A major challenge was the changing team dynamics and constantly changing personnel. This threatened the routine aspect of interpersonal communication, and simple communication techniques such as getting to know the team were not used when the team assembled. Creating a culture of psychological safety was identified as an enabler to team members speaking up to communicate a problem or clarify information. However, some members found speaking up in a critical situation challenging and were reluctant to do so because of fear of conflict or looking incompetent or intrusive.

### Step 3: Implementation strategies to address barriers and enhance enablers to TTT use in trauma resuscitations

#### Intervention functions

Seven intervention functions were selected: ‘Education’, ‘Training’, ‘Environmental restructuring’, ‘Modelling’, ‘Enablement’, ‘Persuasion’ and ‘Incentivisation’. Both the teaching faculty who delivered the training and members of the multidisciplinary trauma team were the targets for behaviour change. The BCTs selected to guide the implementation strategies are outlined in Table 3.

Innovative educational strategies are needed to support the multidisciplinary trauma team to learn ‘teaming’, which is how to practise teamwork in a ‘flash’ team. Persuasion and enablement are required to optimise the team’s intention to use teamwork skills in clinical practice. The modelling of expert team behaviours by senior clinicians is necessary to promote uptake by team members to help create and sustain effective resuscitation teams. Environmental restructuring is needed to prompt the trauma team to gain adequate control of the resuscitation bay in an emergency, specifically with respect to space, noise and crowd control. Incentivisation by managers is required to build a climate of psychological safety where teamwork is recognised and encouraged.

#### Behaviour change techniques and modes of delivery

BCT and modes of delivery to implement the seven interventions are presented in Table 3. These include changes to the instructional design of the simulated multidisciplinary TTT programme to teach ‘teaming’ [27]. This may be achieved through changing the existing programme to include education modalities such as spaced learning [28], contextual learning [29], rapid cycle deliberative practice of non-technical skills [30] and the use of feedback and debriefing techniques [31]. It also involves the teaching of the ‘Zero Point Survey [32]’ which is a structured approach to incorporate teamwork factors into the flow of trauma resuscitation. Standardising the resuscitation environment was also identified as an enabler to teamwork. Standardising equipment layout and checking procedure may enhance team performance by optimising the order and efficiency of the resuscitation. It can also prompt the recognition of unpredictable events. The use of teamwork cues such as posters, trauma algorithms and checklists were also identified to reduce unwarranted variations in patient care when there is a sudden change in the patient or environment (equipment failure, arrival of multiple traumas, too many non-participants in the resuscitation bay). This structured approach also applies to communication in high stress resuscitations where structure delivers clear unambiguous information.

#### Policy categories

Four policy categories were nominated to deliver intervention functions, ‘guidelines’, ‘regulation’, ‘service provision’ and “communication/marketing” and are presented in Table 3. The policy category recommends that a governance committee, comprising of a medical director and nurse manager from all the services involved in trauma care be set up to regulate compliance with the changes to practice recommended by TTT and manage feedback from clinicians and other stakeholders. A policy document will be created that mandates all resuscitation teams to use trauma treatment protocols (i.e. trauma care algorithms) when resuscitating major trauma patients. The use of digital platforms (e.g. email, hospital broadcast, smartphone apps, dashboards, blogs, podcasts) will market the TTT programme and expected outcomes to the emergency trauma community across the hospital. The development of a performance measurement programme will govern service provision by establishing trauma performance standards linked to teamwork. This data will be presented to key stakeholders at the governance meeting. In addition, all trauma team members will have to complete TTT before joining the team and faculty development programme could be considered to provide ongoing simulation instructor development. The resources available to support these policy categories will be allocated within the current education and service provision budgets.

## Discussion

This study has increased our understanding of what will enable teamwork in real life trauma resuscitation events. Educational efficiency and contextualised local implementation strategies are key elements to close the current gap that exists in the literature around the training impact on team performance in resuscitation events.

### Innovative educational strategies

The current TTT programme needs refinement to ensure that it is fit for purpose, i.e. training participants to work in spontaneously created trauma teams with constantly changing membership. There is a need to teach participants how to team [27]. TTT needs to focus on standard teamwork skills (e.g. leadership and followership, communication, situational awareness, resource management) but also integrate facets specific to the function of a ‘flash team’ such as integrating perspectives from a range of disciplines, communicating despite the different mental models that accompany different areas of expertise and being able to manage the inevitable conflicts that arise when people work together in a crisis situation.

Participants wanted TTT to be repeated, rather than provided once, so that the effects could be sustained. The current schedule of a 1-day TTT needs to be supplemented with spaced practice [28], which offers shorter, more frequent learning sessions repeated at regular intervals. Introducing shorter learning sessions such as training stations and refresher events scheduled at frequent intervals will help participants retain teamwork skills.

Participants reported difficulty with the fidelity of simulation as it did not mirror the clinical setting. This is a known limitation of simulation [6]. Greater effort is needed to use training experiences that apply to participants’ real-clinical context. This can be achieved by targeting both learner and environmental factors. Educational strategies include, but are not limited to, ‘in-situ’ simulations which are teaching experiences conducted within the physical space where a resuscitation would be conducted. Enhanced realism for team training using high fidelity simulation can also be achieved by using actors, to more closely resemble an actual patient. Training in resuscitation decision-making could be addressed by increasing the cognitive load and stress factors in the programme. Utilising real clinical events as learning opportunities by debriefing with the team after a resuscitation also provides opportunities for learning [31].

Whilst a mastery learning model is frequently used to develop technical skills, we advocate the use of simulation with rapid cycle deliberate practice to develop teamwork skills [30]. This would involve the teaching of teamwork skills and frameworks such as the Zero Point Survey [32], which is a structured approach to using teamwork factors in a resuscitation. By using repetition and feedback, this educational method provides opportunity to practise the nontechnical elements of resuscitation [33].

### Cultivating a culture of teaming

In our study, the need to focus on cultivating a teamwork culture was identified as the trauma resuscitation setting which was often the place where different specialty and disciplinary cultures clashed. Team members experienced specialty and discipline rivalry, sense of entitlement or differing style of communication as barriers to teamwork in clinical practice. Teamwork skills were not reinforced in the clinical resuscitation environment. In addition, participants reported feeling hesitant to speak up in front of people they did not know very well. The implementation strategy to address changing team culture is multifaceted. Engaging sponsorship from senior leaders such as consultants, heads of department and nurse managers is imperative [34, 35]. Senior clinicians and managers need to model teamwork through demonstrations of behaviour that promotes good interpersonal relations with all team members, regardless of discipline or speciality. Careful use of performance feedback and audits by managers may also be important for persuasion, (through recognition) and as an incentive (through goal setting and recognition) leading to potentially important changes in team culture.

### Environmental restructuring

Changes to environmental factors are needed to achieve sustainable implementation of TTT outcomes into practice. Whilst resuscitations can be unpredictable, the period before first patient contact can augment teamwork if it is used to prepare the environment, team and equipment. It can decrease the likelihood that the resuscitation will be compromised by the unpredictability of a crisis situation, equipment failure and excessive noise or overcrowding. A checklist may be devised to ensure that equipment is available, working and located correctly for every patient, every time. Systems for equipment display will be revised with the aim of ensuring resuscitation equipment is ergonomically displayed.

The inclusion of visual prompts into the resuscitation bay will help to direct time-critical interventions. A stop clock placed on a clearly visible wall in the resuscitation bay will assist in monitoring time critical intervals. Trauma care algorithms will be displayed on a screen to guide the resuscitation process but also as a prompt for the team to share a common approach and language. Similarly, it will prompt team members to question a deviation from standard care. Structured debriefing post resuscitation events is also recommended to ensure that debriefing becomes part of the team culture which is known to improve team management of resuscitation events [31].

## Limitations and strengths

The main strength of this study is the systematic approach to defining, in behavioural terms, the problem of operationalising a training impact from TTT into the context of resuscitating critically injured patients in the clinical setting. Whilst the study was founded on the TDF and the expertise of experienced clinicians, the selection of intervention strategies was subject to some interpretation and this is a limitation. This limitation could be enhanced through inclusion of ethnography, which could focus on flash teams in action and provide insight into the interactions among flash teams in clinical practice. Another limitation is that this is a single-centre study, and hence, the results may not be generalisable to other trauma centres or trauma teams. Future studies may also lend themselves to a stepped wedge design, where the intervention could be rolled out sequentially across a number of major trauma centres over a number of time periods to facilitate a more comprehensive evaluation of the training.

## Conclusion

This study offers a framework for deductively employing the theoretical domains framework, behaviour change wheel and behaviour change techniques to assess and develop intervention strategies to improve the functioning of trauma resuscitation teams.

## Abbreviations

APEASE: Affordability, Practicability, Effectiveness and cost-effectiveness, Acceptability, Side-effects or safety and Equity
BCT: Behaviour change technique
BCW: Behaviour change wheel
TDF: Theoretical domains framework
TTT: Trauma team training
